# Supplementary material for: The Antitumor Effect of Lipophilic Bisphosphonate BPH1222 in Melanoma Models: The Role of the PI3K/Akt Pathway and the Small G Protein Rheb
Source: Int J Mol Sci. 2019 Oct 3;20(19):4917. doi: 10.3390/ijms20194917 (PMC6801414; doi:10.3390/ijms20194917)
Supplement: Supplementary file 1 [file ijms-20-04917-s001.pdf]

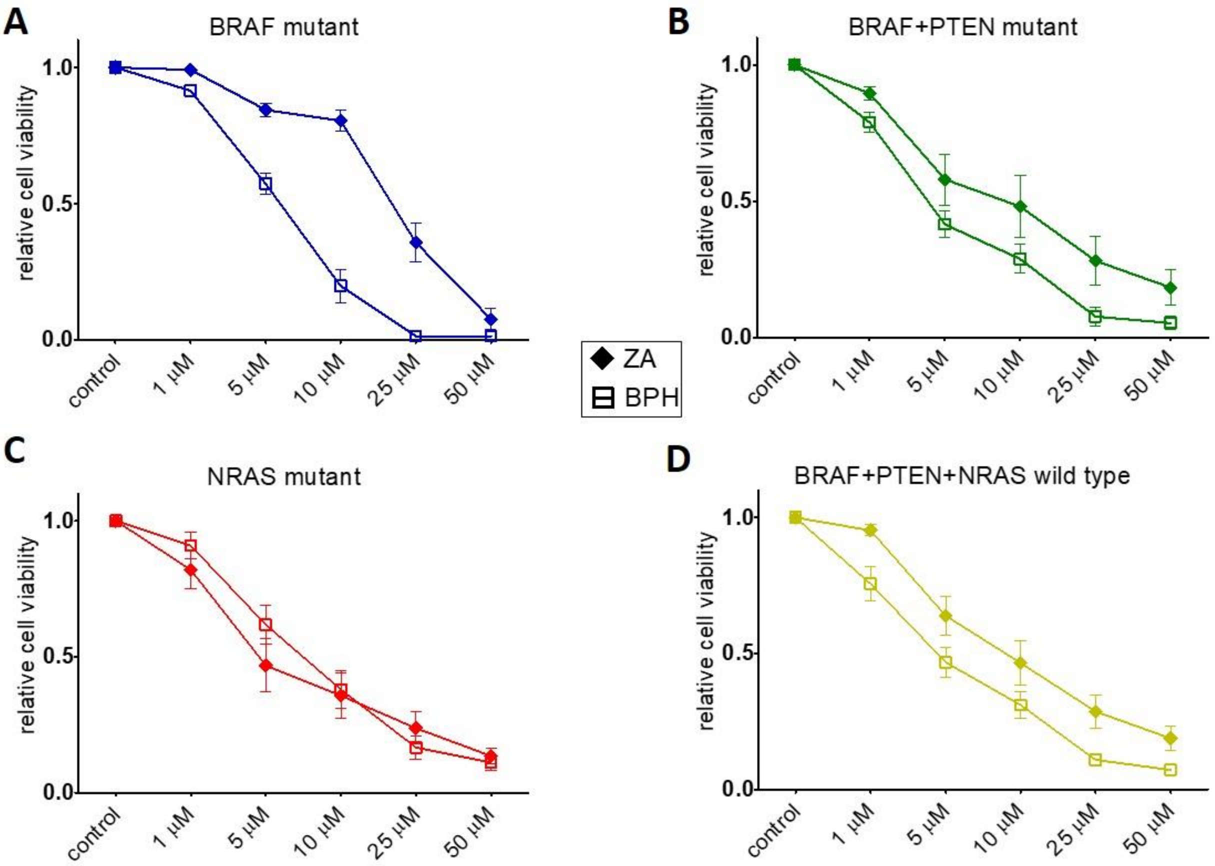

Figure S1. Average of the relative cell viability results upon treatment with ZA or BPH for 72h, grouped by the mutational status of the cell lines. The differences are most pronounced in the blue marked BRAF mutant group, however, NRAS mutant cell lines were more sensitive to ZA. Data are average +/- SEM from at least 6 independent measurements.

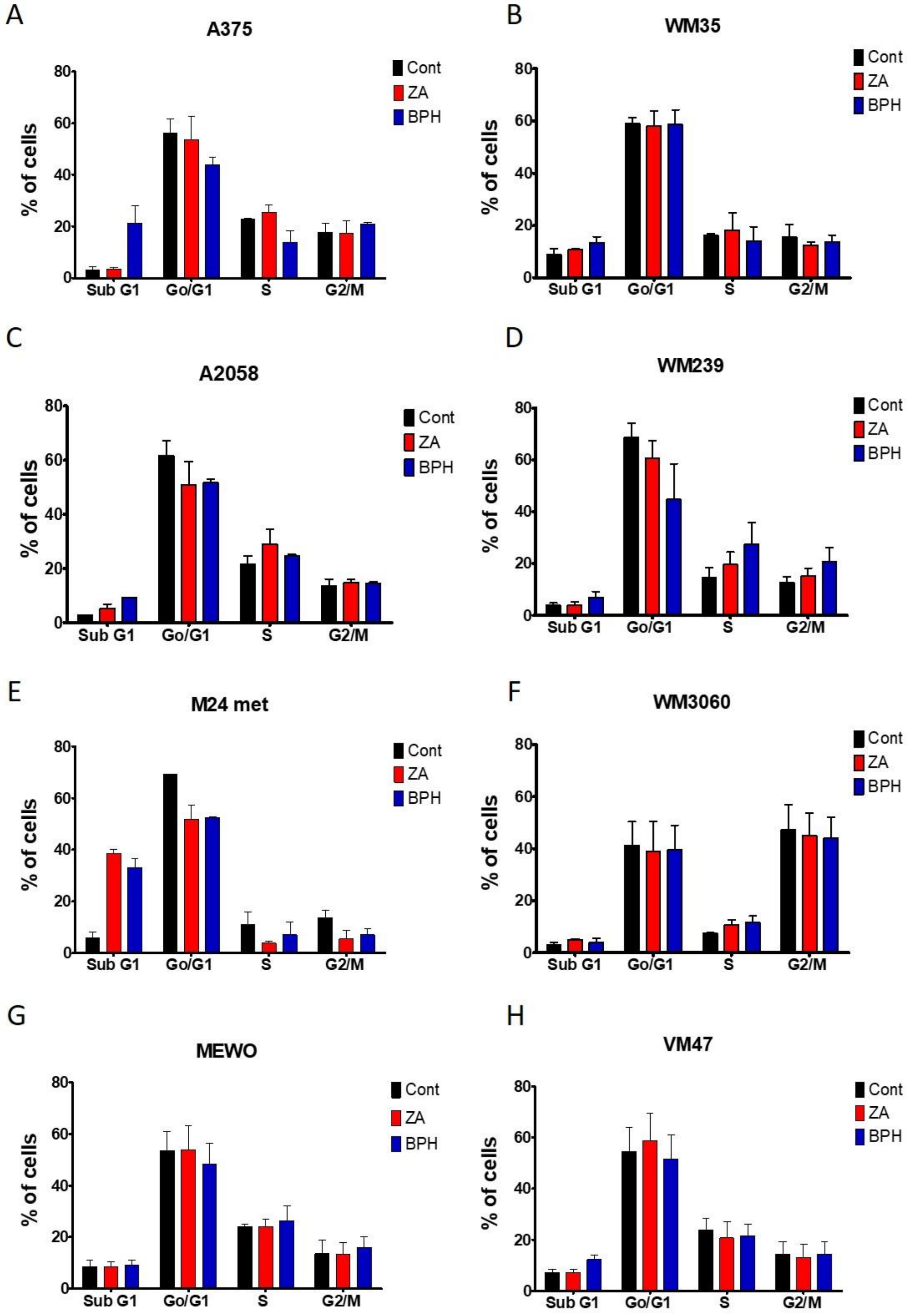

Figure S2. Cell cycle analysis upon treatment with 10μM ZA or BPH was carried out for 72 h. Data are shown average +/- SD from two to three independent experiments.

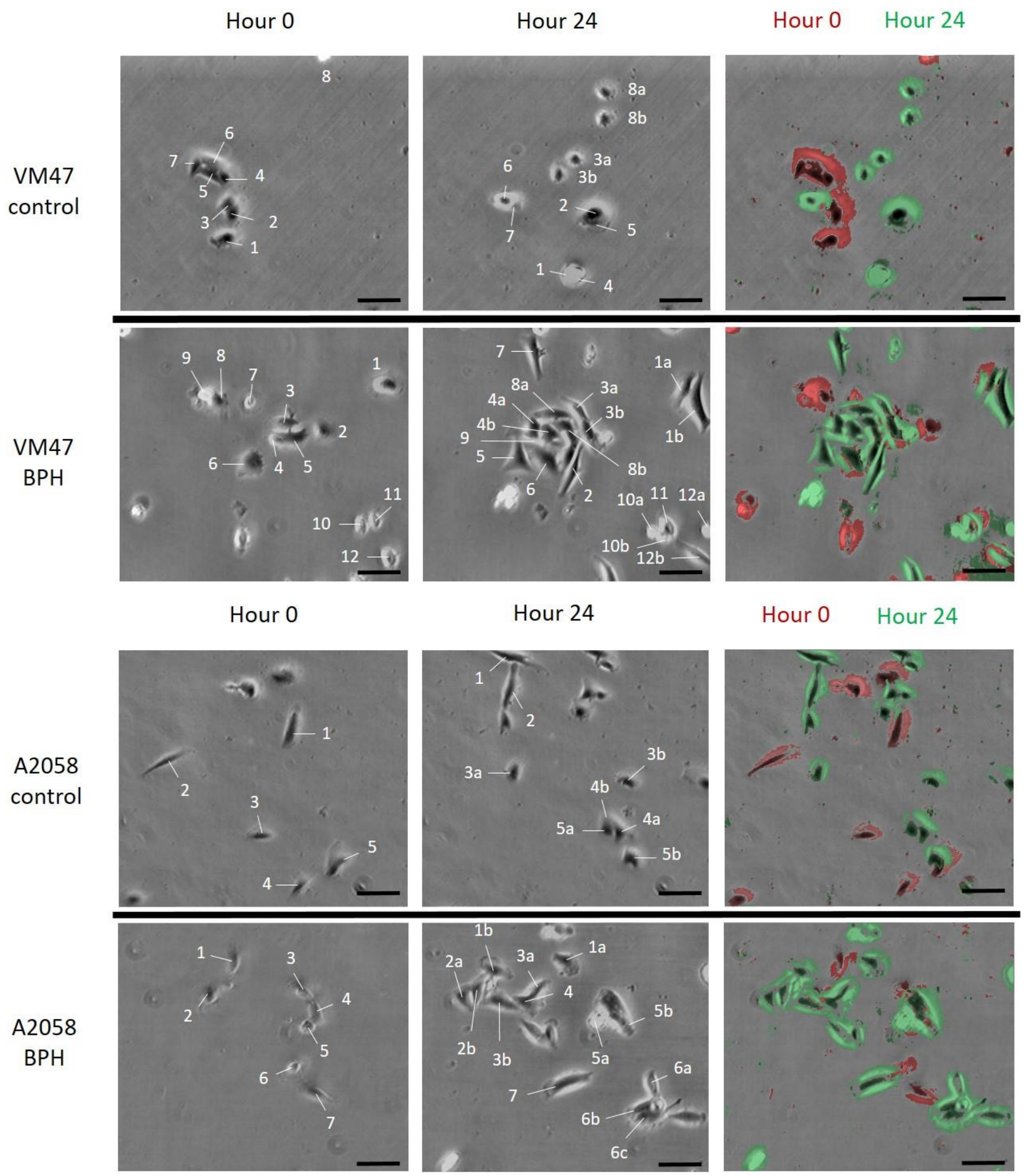

Figure S3. Representative pictures from the videomicroscopy measurements of A2058 and VM47 (the two most sensitive cell lines in terms of migration) at 0- and 24-hour time point. Pictures are a part of the original pictures, presenting a small group of cells. While control cells have a higher displacement, cells upon treatment with BPH do not migrate far away. Numbers mark the same cells at hour 0 and hour 24. Numbers with 'a' or 'b' designate the daughter cells of the given cell. Cells without numbers mean that those are not on both of the pictures, as either came from out of the picture or went out from the picture during 24 hours. Pictures on the third column show the merged images of hour 0 (red cells) and hour 24 (green cells). Scale bar means 40μm.

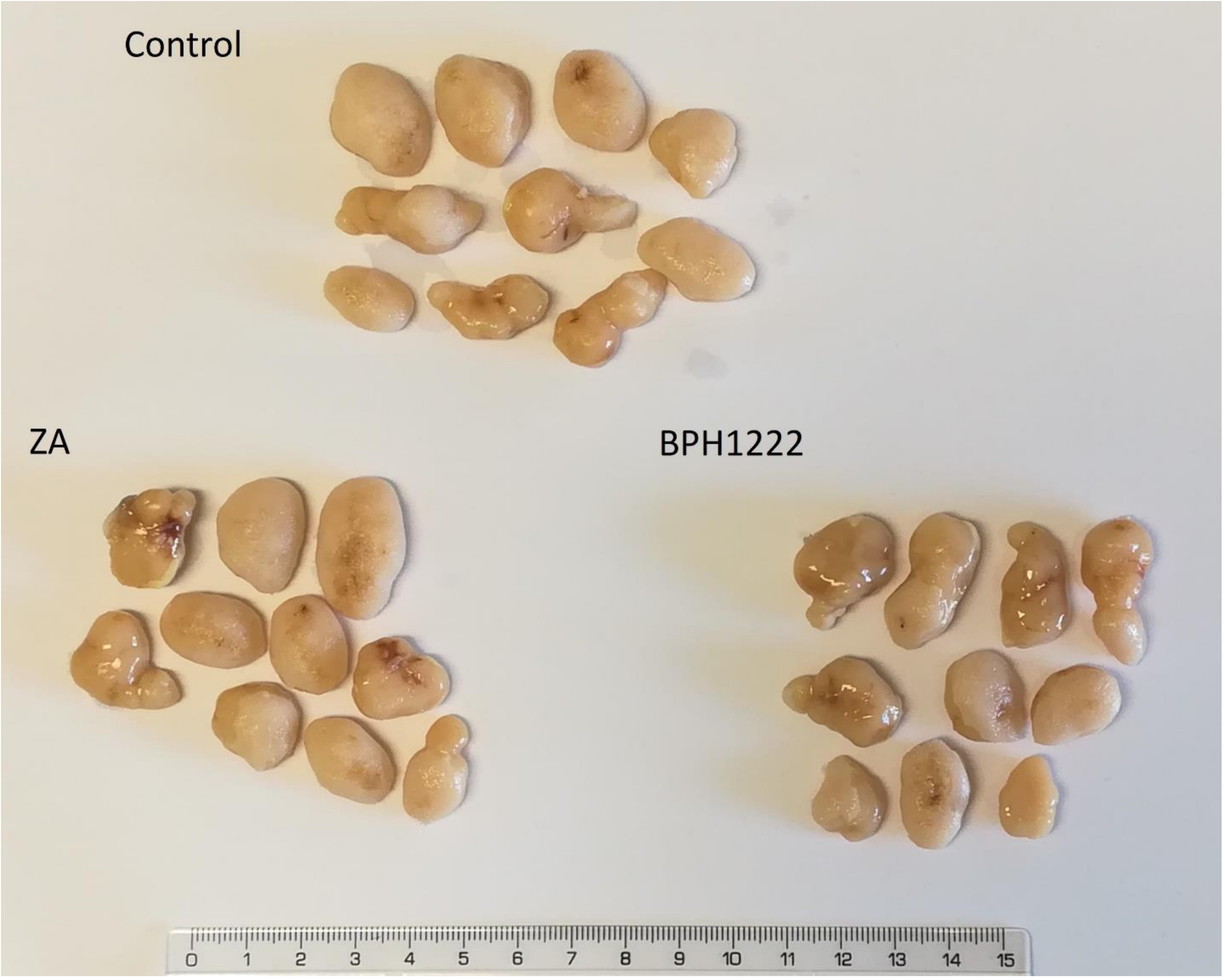

Figure S4. The dissected tumors from the *in vivo* experiment.

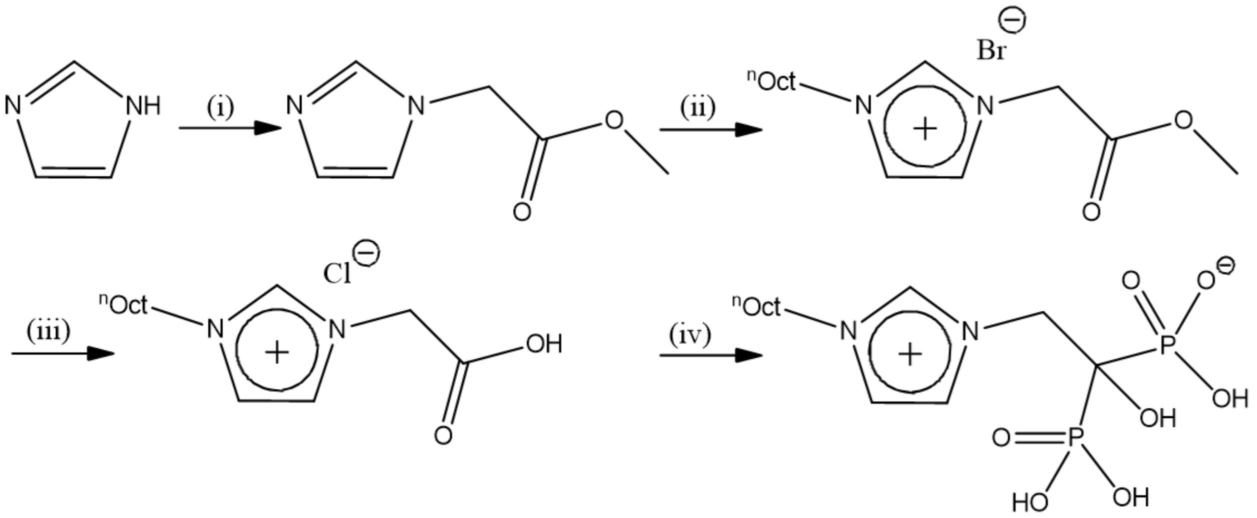

Figure S5. Isolation steps of BPH1222 during chemical synthesis. Yields of isolated products are in brackets:

(i) K_2CO_3, NaI, acetone, reflux, followed by column chromatography on silica, eluent dichloromethane - ethanol 9:1 (39%) [1]

(ii) 1-bromooctane, ethyl acetate, reflux, followed by column chromatography on silica, eluent ethyl acetate - methanol 9:1 and dichloromethane - ethanol 9:1 (45%)

(iii) cc. HCl – H_2O 1:1, reflux, evaporation to dryness, washing with acetone (90%)

(iv) H_3PO_3, $POCl_3$, toluene, 95°C, reflux with cc. HCl – H_2O 1:1, evaporation to dryness, recrystallization from water – isopropyl alcohol (70%) [2]

^{n}Oct means: (CH_3)-$(CH_2)_7$

1. Hack, S.; Worlein, B.; Hofner, G.; Pabel, J.; Wanner, K.T. Development of imidazole alkanoic acids as mGAT3 selective GABA uptake inhibitors. *Eur J Med Chem* **2011**, *46*, 1483-1498, doi:10.1016/j.ejmech.2011.01.042.
2. Zhang, Y.; Zhu, W.; Liu, Y.L.; Wang, H.; Wang, K.; Li, K.; No, J.H.; Ayong, L.; Gulati, A.; Pang, R., et al. Chemo-Immunotherapeutic Anti-Malarials Targeting Isoprenoid Biosynthesis. *ACS medicinal chemistry letters* **2013**, *4*, 423-427, doi:10.1021/ml4000436.

Table S1. Summary table of data based on the mutational status of the cell lines. First column: percentage of living cells compare to control based on long term (10 days) clonogenic assay results with 1μM treatment. Second column: relative migration decrease (-) or increase (+) upon treatment compared to the control. Third column: relative spheroid volume compared to control with 2μM treatment at the sixth day. Four of the eight cell lines (WM35, WM239, WM3060, MEWO) were not able to generate spontaneously spheroids. Fourth-fifth columns: protein (Akt, Erk) activation changes (-decrease, + increase) or no change (0) upon treatment.

Mutation	Cell name	Long term relative viability 1μM		Relative migration		3D spheroid 2μM 6 days		Western blot - pAkt		Western blot - pErk	
		ZA	BPH	ZA	BPH	ZA	BPH	ZA	BPH	ZA	BPH
BRAF	A375	78%	3,9%	+14%	-4%	74%	9,2%	0	-	0	0
	WM35	72%	16%	-1%	-18%	not applicable		-	-	0	0
BRAF+PTEN	A2058	64%	20%	-18%	-32%	85%	80%	0	0	0	0
	WM239	27%	13%	+16%	-23%	not applicable		0	0	0	0
NRAS	M24met	5,1%	18%	+10%	-5%	51%	56%	-	-	-	-
	WM3060	47%	44%	+20%	+34%	not applicable		0	+	0	0
WT	MEWO	29%	18%	+38%	+14%	not applicable		0	0	0	0
	VM47	51%	11%	+8%	-51%	92%	71%	0	-	0	+
